# Tuberculosis Among Native Hawaiian and Other Pacific Islander Persons: United States and U.S.-Affiliated Pacific Islands, 2010–2019

**DOI:** 10.1089/heq.2022.0065

**Published:** 2022-06-27

**Authors:** Molly Deutsch-Feldman, Yuri P. Springer, Derrick Felix, Clarisse A. Tsang, Richard Brostrom, Maryam Haddad

**Affiliations:** ^1^ Division of Tuberculosis Elimination, National Center for HIV, Viral Hepatitis, STDs, and TB Prevention, Centers for Disease Control and Prevention, Atlanta, Georgia, USA.; ^2^ Epidemic Intelligence Service, Centers for Disease Control and Prevention, Atlanta, Georgia, USA.

**Keywords:** U.S.-Affiliated Pacific Islands, tuberculosis

## Abstract

**Background:**

In recent years, tuberculosis (TB) incidence in the United States has declined overall but remained high among Native Hawaiian and Other Pacific Islander (NH/PI) persons. Few studies have examined the epidemiology of TB among NH/PI persons, particularly in the U.S.-Affiliated Pacific Islands (USAPI). We describe TB incidence and characteristics of NH/PI patients during 2010–2019.

**Methods:**

We used data from the National Tuberculosis Surveillance System to characterize TB cases reported among NH/PI persons born in the 50 U.S. states (defined to include District of Columbia) and the USAPI. We calculated annual TB incidence among NH/PI patients, stratified by place of birth (U.S. states or USAPI). Using Asian persons born outside the United States—persons historically grouped with NH/PI persons as one racial category—as the reference, we compared demographic, clinical, and socio-behavioral characteristics of NH/PI TB patients.

**Results:**

During 2010–2019, 4359 TB cases were reported among NH/PI patients born in the U.S. states (*n*=205) or the USAPI (*n*=4154). Median annual incidence per 100,000 persons was 6.5 cases (persons born in the U.S. states) and 150.7 cases (persons born in the USAPI). The proportion of TB patients aged <15 years was higher among NH/PI persons (U.S. states: 54%, USAPI: 24%) than among Asian persons born outside the United States (1%).

**Conclusions:**

TB incidence among NH/PI persons is high, particularly among persons born in the USAPI, emphasizing the need to enhance TB prevention strategies in these communities. Interventions should be tailored toward those who experience the highest risk, including NH/PI children and adolescents.

## Introduction

Although overall tuberculosis (TB) incidence has declined in the United States in recent years, incidence among Native Hawaiian and Other Pacific Islander (NH/PI) persons residing in the United States at the time of their TB diagnosis has remained high.^1^ In addition, TB incidence in the U.S.-Affiliated Pacific Islands (USAPI) is higher than in the United States.^1^ Some NH/PI populations experience higher rates of infectious diseases (such as sexually transmitted diseases and Hansen's disease),^2–4^ diabetes, and infant mortality, and they have lower life expectancy compared with non-Hispanic White persons.^5,6^

Similarly, social determinants of health, including educational attainment, income, and health insurance coverage, are all lower among NH/PI persons than among non-Hispanic White persons.^5,7,8^ Despite these inequities, a few epidemiologic studies have examined TB among NH/PI persons, and TB in the USAPI specifically.^9,10^ Additional work to characterize the disproportionately high rates of TB disease among NH/PI persons is needed to inform tailored public health interventions that reduce incidence in this population.^1,7^

Given the differences in TB incidence between the USAPI and the 50 U.S. states, understanding the epidemiology of both regions is important for designing and implementing effective TB control measures. In addition, TB disease in the United States is strongly influenced by place of birth.^1^ The USAPI (comprising six areas: Guam, the Commonwealth of the Northern Mariana Islands, American Samoa, the Federated States of Micronesia, the Republic of the Marshall Islands, and the Republic of Palau) conduct TB surveillance in partnership with the Centers for Disease Control and Prevention (CDC). However, detailed published data regarding TB in the USAPI are limited; to our knowledge, no studies of TB disease in NH/PI populations have explored differences in TB epidemiology by place of birth.

In this analysis, we compared disease incidence associated with, and demographic and clinical characteristics of, NH/PI TB patients born in the 50 states or DC and those born in the USAPI. We stratified our analysis by place of birth (U.S. states or USAPI) to understand TB epidemiology within populations with substantially different baseline risks for TB.

## Materials and Methods

### National Tuberculosis Surveillance System

We analyzed TB case data from the National Tuberculosis Surveillance System (NTSS), a database maintained by the CDC. NTSS reporting areas comprise all 50 states, the District of Columbia (DC), New York City, Puerto Rico, the U.S. Virgin Islands, and the six USAPI regions. Each reporting area submits case reports containing standardized demographic and clinical data elements for each verified TB case to NTSS.^1^

This project was determined not to be human subjects research by the CDC and did not require approval by an institutional review board, because data were collected and analyzed as part of routine public health surveillance.

### Population

Our analyses included a subset of all incident TB cases reported to NTSS during January 1, 2010–December 31, 2019 and counted in any of the 50 states or Washington, DC or any of the six USAPI areas.

The TB patients were categorized as Hispanic (regardless of patient race) or non-Hispanic based on patient self-reported ethnicity.^11^ Cases in patients not reporting Hispanic ethnicity were then further categorized by self-reported race into standard federal race categories: American Indian/Alaska Native, Asian, Black/African-American, NH/PI, White, and multiple race.^11^ Following previous studies of TB among NH/PI persons,^9,12^ persons reporting ≥1 race or Hispanic ethnicity were not included in the NH/PI category.

The analysis population included and was determined by categorizing cases as one of four analytic groups using patient self-reported race, ethnicity, and place of birth: non-Hispanic single-race NH/PI TB patients born in the 50 states or DC (hereafter, “NH/PI 50 states”), non-Hispanic single-race NH/PI patients born in the USAPI (“NH/PI USAPI”), non-Hispanic single-race Asian patients born in Asia^†^, (“Asian”), and non-Hispanic single-race White patients born in the 50 states or DC (“White”).

Because Asian persons born in the U.S. states constituted <5% of all Asian TB patients, and experience substantially lower TB risk,^1^ we excluded these persons. We relied on patient-reported county of birth to determine place of birth. Persons born in Guam and the Commonwealth of the Northern Mariana Islands are considered “U.S.-born,” whereas persons born in the remaining four USAPI jurisdictions are not.^13^

### Statistical analyses

We enumerated annual incidence (cases per 100,000 persons) by analytic group using estimates of annual group population size obtained from multiple data sources (e.g., U.S. Census Bureau's American Community Survey, U.S. Census Bureau's International Database, United Nations World Population Prospects).^14–16^ We calculated annual incidence for each of the four groups described earlier as well as for each of the USAPI regions. These latter calculations included all cases in NH/PI patients who were born in the USAPI and reported as a verified TB case anywhere in the USAPI. We also calculated incidence for the NH/PI 50 states analytic group among the U.S. Department of Health and Human Services (HHS) Region.^17^ Additional methods regarding the population data sources and incidence calculations are provided in Supplementary File S1.

We compared the annual TB incidence among analytic groups by calculating the incidence rate ratio (IRR) and associated 95% confidence intervals (CI). Our primary comparisons used the Asian analytic group as the reference. We used the White analytic group as a reference for secondary analyses (additional information is provided in Supplementary File S1). We calculated CIs using the normal approximation (Wald) method if the numerator for any group calculation was >10 and otherwise using a bootstrap method with 10,000 replicates.

We compared the distributions of demographic and clinical characteristics of TB disease between the NH/PI analytic groups and the Asian group by using chi-squared tests or Fisher's exact tests (for proportions with numerators <10). We further analyzed selected clinical and sociodemographic characteristics of disease among patients aged ≥15 by comparing the prevalence of each patient characteristic among groups using a bivariate prevalence ratio (PR) and associated 95% CI.

Additional details regarding the definitions of these characteristics are available in Supplementary File S1. As with the incidence comparisons, we used the Asian analytic group as the reference for our primary analyses. We calculated the 95% CIs as described earlier. We defined statistical significance using an alpha level of 0.05. Additional details regarding the methods for these calculations are provided in Supplementary File S1.

## Results

### Case counts

During 2010–2019, 101,209 incident TB cases were reported to NTSS. Of these, 4,359 (4%) were in NH/PI patients born in the U.S. states or USAPI: 205 (4.7%) in NH/PI 50 states patients and 4,154 (95.3%) in NH/PI USAPI patients (Table 1). Three hundred forty-one TB cases in NH/PI patients born in countries other than the United States or the USAPI were excluded from the analyses (Supplementary Table S1). The Asian and White analytic groups contained 29,901 (30% of total) and 10,119 (10% of total) cases, respectively.

**Table 1.** Demographic and Clinical Characteristics of Non-Hispanic Native Hawaiian and Other Pacific Islander and Asian Persons with Tuberculosis Disease, 2010–2019

Characteristic	*n* (%)^a^			
	NH/PI 50 states^b^	NH/PI USAPI^b^	Asian^b^	White^b^
Total number of cases	205	4154	29,901	10,119
Sex				
Male	111 (54)	2157 (52)^*^	17,089 (57)	6944 (69)
Female	94 (46)	1997 (48)^*^	12,812 (43)	3175 (31)
Age (years)				
0–4	69 (34)^*^	438 (11)^*^	122 (<1)	188 (2)
5–14	41 (20)	558 (13)	247 (1)	84 (1)
15–24	29 (14)	707 (17)	2598 (9)	335 (3)
25–44	30 (15)	1165 (28)	8899 (30)	1649 (16)
45–64	22 (11)	1008 (24)	9000 (30)	4216 (42)
≥65	14 (7)	277 (7)	9033 (30)	3646 (36)
Ethnicity^c^				
Chamorro	<5 (1)	180 (4)		
Fijian	<5 (<1)	<5 (0)		
Kiribati	<5 (0)	<5 (<1)		
Marshallese	25 (12)	1967 (47)		
Micronesian	34 (17)	1555 (37)		
Native Hawaiian	24 (12)	<5 (<1)		
Palauan	<5 (1)	94 (2)		
Samoan	15 (7)	21 (1)		
Tongan	14 (7)	<5 (0)		
Other	11 (5)	27 (1)		
Not reported	78 (38)	307 (7)		
Clinical characteristics				
Diabetes mellitus at time of diagnosis	20 (10)^*^	832 (20)^*^	6506 (22)	1253 (12)
HIV positive at time of diagnosis^d^	<5 (0)	<5^*^(<1)^*^	359 (1)	296 (3)
End-stage renal disease or chronic renal failure at time of diagnosis	5 (2)	44 (1)^*^	904 (3)	169 (2)
Immunosuppressed at time of diagnosis^e^	7 (3)	41 (1)^*^	1794 (6)	1088 (11)
Diagnostic criteria				
Smear positive^f^	37 (35)	1277 (36)^*^	10,364 (40)	4092 (49)
Culture positive^f^	61 (58)	1600 (49)^*^	16,866 (66)	5797 (70)
Disease status				
Any pulmonary disease	171 (83)^*^	3453 (83)^*^	22,950 (77)	8718 (86)
Any extrapulmonary disease	73 (36)	960 (23)^*^	10,077 (34)	2097 (21)
Cavitary pulmonary disease^g^	26 (13)^*^	921 (22)^*^	7167 (24)	3597 (36)
Miliary disease^h^	11 (6)^*^	144 (4)^*^	859 (3)	349 (4)
Meningeal disease	8 (4)^*^	8 (<1)^*^	371 (1)	109 (1)
Completed TB treatment^i^	166 (94)	2953 (90)	23,289 (89)	8159 (86)

This table includes all single-race, non-Hispanic NH/PI persons and single-race, non-Hispanic Asian persons born in Asia diagnosed with TB disease between 2010 and 2019.

^a^ Values <5 are presented as “<5.”

^b^ NH/PI 50 states includes all single-race NH/PI persons who reported being born in the 50 U.S. states or Washington, DC; NH/PI USAPI includes all single-race NH/PI persons who reported being born in the USAPI; Asian includes all single-race Asian persons who reported being born in Asia; White includes all single-race White persons who reported being born in the 50 U.S. states or Washington, DC.

^c^ Micronesian includes Carolinian, Chuukese, Kosrean, Micronesian, Pohnpeian, and Yapese; Chamorro includes Chamorro, Guamanian, and Saipanese; Other includes Mariana Islander, Melanesian, New Herbides, Other Pacific Islander, Polynesian, Solomon Islander, and Tokelauan.

^d^ 2011–2019 only.

^e^ Includes patients who had immunosuppression due to either a medical condition or medication, or immunosuppressive therapy, excluding diabetes mellitus, end-stage renal disease, HIV/AIDS, and patients who had recently received, or were receiving, TNF-α antagonist therapy at the time of TB diagnosis.

^f^ Proportions calculated only among patients with available test results.

^g^ Includes only persons with any pulmonary TB and any evidence of cavitation on chest radiograph or computerized tomography scan.

^h^ Any evidence of miliary disease on chest radiograph.

^i^ Numbers and percentages are based on patients with complete outcome data with a 2 year lag (i.e., cases reported during 2010–2018) so that outcome data could be documented in the 2010–2020 dataset.

^*^ Indicates *p*<0.05 for the chi-squared test or Fisher's exact test comparing the indicated proportion with that of the Asian analytic group. For the age variable, group-wise comparisons were performed.

NH/PI, Native Hawaiian and Other Pacific Islander; TB, tuberculosis; USAPI, U.S.-Affiliated Pacific Islands.

### Incidence

**FIG. 1.**
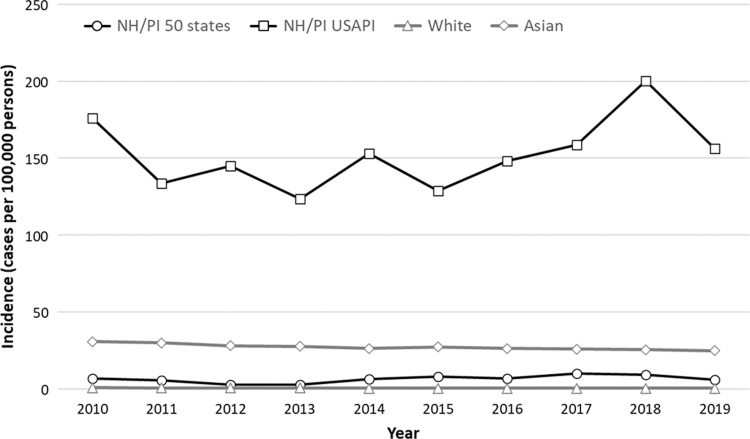
Annual incidence of TB disease (cases per 100,000 persons) among non-Hispanic, single-race NH/PI, Asian and White persons by place of birth, 2010–2019. Incidence calculated as quotient of (1) number of NH/PI TB patients born in the U.S. states and estimated number of NH/PI persons born in the U.S states, (2) number of NH/PI TB patients born in the USAPI and estimated number of NH/PI persons born in the USAPI, (3) number of White TB patients born in the U.S. states and estimated number of White persons born in the U.S states, and (4) number of Asian TB patients born in Asia and estimated number of Asian persons born in Asia. See Supplementary Text for additional details regarding population denominator estimates. Note: multiple mass screening events to identify cases of active TB were conducted in the Republic of the Marshall Islands starting in 2017. NH/PI, Native Hawaiian and Pacific Islander; TB, tuberculosis; USAPI, U.S.-Affiliated Pacific Islands.

Median annual incidence (cases per 100,000 persons) in the NH/PI USAPI group was 150.7, (range=123.3 [2013]–200.2 [2018]) (Fig. 1, Supplementary Table S2) and 6.5 for the NH/PI 50 states group (range=2.6 [2013]–10.1 [2017]). Compared with the Asian group, incidence in the NH/PI USAPI group was consistently higher, ranging from an IRR of 4.5 (95% CI=4.0–5.0) (Supplementary Table S2) in 2011 to 7.9 (95% CI=7.2–8.6) in 2018. Incidence was consistently lower for the NH/PI 50 states group compared with the Asian group. The IRR ranged from 0.1 (0.0–0.2) in 2013 to 0.4 (95% CI=0.3–0.5) in 2018.

Compared with the White group, incidence was consistently higher for both NH and PI analytic groups. The IRRs for the NH/PI USAPI group ranged from 196.1 (95% CI=174.4–220.5) in 2011 to 464.7 (95% CI=417.1–517.7) in 2018. The IRRs for the NH/PI 50 states group ranged from 4.1 (95% CI=1.5–7.2) in 2012 to 24.3 (95% CI=17.2–34.2) in 2017.

**FIG. 2.**
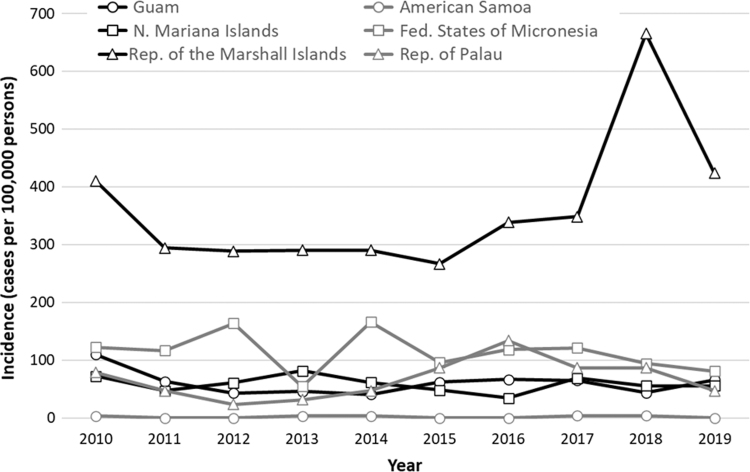
Crude annual TB incidence (per 100,000 persons) among NH/PI persons with TB disease reporting being born in the USAPI by region, 2010–2019. Incidence proportions calculated for each region/year as the quotient of the number of TB cases reported among NH/PI persons reporting being born in any of the six USAPI regions and estimated number of NH/PI persons reporting being born in each region (see Supplementary Text for additional details regarding population estimates). Note: multiple mass screening events to identify cases of active TB were conducted in the Republic of the Marshall Islands starting in 2017.

**FIG. 3.**
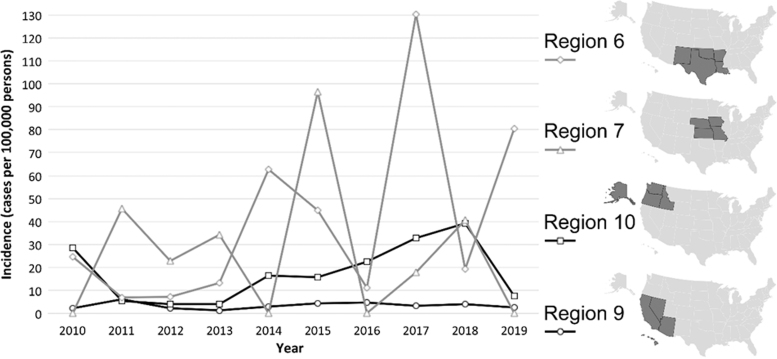
Crude annual TB incidence (per 100,000 persons) among NH/PI persons with TB disease reporting being born in the 50 States among the four Department of Health and Human Service regions with highest median incidence, 2010–2019. Incidence proportions calculated for each region/year as the quotient of the number of TB cases reported among NH/PI persons reporting being born in the 50 states and estimated number of NH/PI persons reporting being born in each HHS region. See Supplemental Text for information regarding population estimates. Note: the small numerators (<5) for many of these calculations yield highly variable incidence estimates that are not statistically reliable. HHS, U.S. Department of Health and Human Services.

Annual incidence of TB cases reported in the USAPI among the NH/PI USAPI group was consistently highest in the Marshall Islands (median=316.6, range=266.5 [2015]–665.5 [2018]) (Fig. 2). Annual incidence of TB cases reported in the 50 states among the NH/PI 50 states group was highest in HHS Region 6 (median=22.0, range=6.9 [2011]–130.1 [2017]) (Fig. 3).

### Patient characteristics

Among NH/PI 50 states TB patients, 54% were male and 54% were aged ≤14 years; among patients born in the USAPI, 52% were male and 24% were aged ≤14 years (Table 1). Most NH/PI patients (both 50 states and USAPI analytic groups) self-identified as Marshallese or Micronesian (Table 1). The NH/PI USAPI TB patients had a lower proportion of positive sputum culture compared with Asian TB patients (49% vs. 66%, *p*<0.01). The proportion of patients with cavitary disease was lower for both the NH/PI 50 states group (22%, *p*<0.05) and the NH/PI USAPI group (13%, *p*<0.01) compared with Asian TB patients (24%).

Diabetes prevalence among all persons in the NH/PI 50 states and NH/PI USAPI groups was 10% and 20%, respectively. Zero HIV diagnoses were reported among NH/PI 50 state patients and <5 among NH/PI USAPI patients (Table 1).

**FIG. 4.**
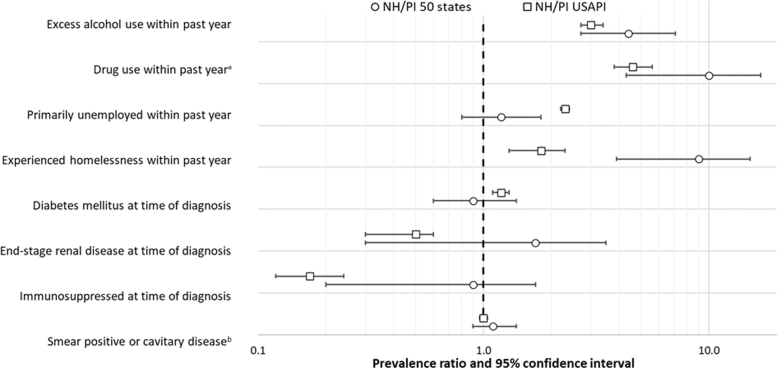
PRs comparing frequency of selected characteristics among NH/PI TB patients (50 states and USAPI) with Asian patients. PRs calculated as the proportion of NH/PI TB patients aged ≥15 years, stratified by place of birth, reporting each characteristic divided by the proportion of Asian TB patients aged ≥15 years reporting each characteristic. See Supplementary Table 1 for additional details. For TB cases reported to NTSS during 2010–2019, patients with unknown or missing information for a characteristic were excluded from PR calculations for that characteristic. The *dashed line* represents a null value of PR=1. ^a^Includes both injection and non-injection drug use. ^b^Smear, sputum smear; restricted patients with pulmonary disease and patients with available information. NTSS, National Tuberculosis Surveillance System; PR, prevalence ratio.

When compared with TB patients age 15 and older in the Asian analytic group, both NH/PI 50 state and NH/PI USAPI patients age 15 and older had a higher prevalence of excess alcohol use within the previous year (PR=4.4, 95% CI: 2.7–7.1; PR=3.0, 95% CI: 2.7–3.4, respectively) and drug use (either injection or non-injection drug use) within the previous year (PR=10.0, 95% CI: 4.3–17.0; PR=4.6, 95% CI: 3.8–5.6, respectively) (Fig. 4, Supplementary Table S3). Patients in both NH/PI analytic groups were also more likely to have experienced homelessness in the previous year (PR=9.0, 95% CI: 3.9–15.2; PR=1.8, 95% CI: 1.3–2.3, respectively) than Asian patients.

Although NH/PI USAPI patients were more likely than Asian patients to report unemployment in the previous year (PR=2.3, 95% CI: 2.2–2.4), NH/PI 50 states patients were not (PR=1.2, 95% CI: 0.8–1.8). There were no differences in the prevalence of patients with smear positive or cavitary disease between the NH/PI 50 states patients (PR=1.1, 95% CI: 0.9–1.3) and the NH/PI USAPI patients (PR=1.0, 95% CI: 1.0–1.0), compared with Asian patients.

Relative to TB patients age 15 and older in the White analytic group, NH/PI 50 state and NH/PI USAPI patients age 15 and older were more likely to have diabetes (PR=1.6, 95% CI: 1.1–2.4; PR=2.1, 95% CI: 1.9–2.2, respectively) (Supplementary Fig. S1, Supplementary Table S4) and less likely to be immunosuppressed^‡^ (PR=0.5, 95% CI: 0.1–0.9; PR=0.1, 95% CI: 0.1–0.1, respectively). Although NH/PI USAPI patients were more likely than White patients to report unemployment in the previous year (PR=1.6, 95% CI: 1.5–1.6), NH/PI 50 states patients were not (PR=0.8, 95% CI: 0.6–1.2).

## Discussion

We found a high incidence of TB among NH/PI persons, particularly among patients born in the USAPI. The higher incidence of TB among NH/PI persons born in the USAPI compared with the NH/PI persons born in 50 states highlights the historically high incidence in the USAPI and the need to enhance TB control and prevention efforts in the USAPI. The NH/PI persons composed ∼0.3% of the combined U.S. and USAPI populations during the investigation period^15,18^ yet 4% of reported TB cases. These findings further emphasize the previously reported higher incidence of TB among indigenous populations compared with persons of other racial and ethnic groups^9,10,12^ and the need to address the documented health disparities associated with NH/PI populations.^2–4,6^ In addition, these findings expand on this previous work focused on persons born in the United States^9^ by exploring TB epidemiology among persons born in the USAPI.

Focusing efforts on TB prevention among NH/PI children is critical. The proportions of TB cases among children aged <15 years observed in this analysis were higher among NH/PI 50 states (54%) and NH/PI USAPI patients (24%) than among all persons with TB in the United States (5%).^1^ Children <5 are more likely to develop TB disease once infected and therefore pediatric infections represent an indicator of recent TB transmission.^19,20^ The markedly higher proportion of pediatric NH/PI TB patients supports previous findings of a high level of recent transmission among NH/PI persons living in the United States at the time of their TB diagnosis; more than a third of TB cases among NH/PI persons were attributed to recent transmission in 2019–2020.^1^ This finding also has important implications for TB surveillance. The high proportion of children with TB may contribute to the lower overall proportion of culture-positive cases among NH/PI patients observed here, and in previous studies,^21^ because TB in children is less likely to be culture confirmed.^22^ Fewer culture-confirmed TB cases hamper surveillance efforts based on genomic sequencing, which uses an isolate from culture.^23^

The high incidence of TB among NH/PI persons is particularly notable considering the low prevalence (<1%) of HIV in this population. This finding is in contrast with many other populations globally, where HIV co-infection is a primary driver of TB disease.^24,25^ Instead, the high incidence of TB among NH/PI persons may be driven by higher rates of TB exposure and contributed to by the high prevalence of diabetes in this analysis. We observed an overall diabetes prevalence of 26% among NH/PI TB patients ≥15 years; the U.S. national estimate of diabetes prevalence among single-race NH/PI adults is ∼18%^26^ whereas the prevalence among all adults in the Western Pacific Region of the world (which includes the USAPI) is ∼10%.^27^ As diabetes is associated with more severe TB disease outcomes,^28^ the diabetes comorbidity has important implications for TB control and prevention programs that otherwise generally focus attention and resources on the role of HIV.

Compared with the Asian adult group, the relatively higher prevalence of drug use and excess alcohol use among NH/PI TB adult patients also has important implications for management of TB disease. Similar to diabetes, injection drug use is a known risk factor for progression to TB among persons who are infected.^29^ Persons who use substances can also face challenges when obtaining medical attention and completing TB treatment.^30,31^

The geographic variation we observed points to regions where TB prevention efforts focused on NH/PI persons would be most appropriate. HHS Region 6 (Arkansas, Louisiana, New Mexico, Oklahoma, and Texas), which had the highest median incidence of TB among NH/PI TB patients in the 50 states, has a large population of NH/PI persons, with several counties in which NH/PI persons compose >1% of the population.^32^ These findings further highlight jurisdictions in need of additional TB support for NH/PI communities.

The steep increase in TB cases found in the Republic of the Marshall Islands beginning in 2017–2019 coincides with multiple mass screening events that were conducted starting in 2017.^33,34^ Active case finding might be a useful tool for interrupting the spread of *M.Tb* in areas of high TB incidence with ongoing transmission. In addition, public health partners and local clinical care providers in these areas should consider whether NH/PI persons may benefit from enhanced TB prevention efforts. The U.S. Preventive Services Task Force and CDC recommend routine latent TB infection screening for persons who experience the highest risk, including those who have lived in areas of increased TB prevalence.^35^

This analysis is subject to several limitations. First, the denominator population data used to calculate incidence were drawn from multiple sources, limiting our ability to compare incidence estimates across the regions. Similarly, population estimates for members of racial and ethnic minority groups, such as NH/PI persons, may be less reliable due to smaller population sizes. Second, we excluded NH/PI persons of multiple race, which may have affected our findings. Third, our statistical analysis was restricted to bivariate associations and cannot be interpreted as causal relationships. Lastly, the low number of observations, particularly among the NH/PI 50 states group, yields variable incidence estimates that are subject to yearly fluctuation.

### Health equity implications

The numerous differences in the characteristics of NH/PI TB patients compared with Asian TB patients—two racial groups previously classified as one^36^—demonstrate the importance of selecting an appropriate reference group when conducting epidemiologic analyses. Though the two groups have been disaggregated in NTSS since 2003,^1^ our analysis demonstrates differences in the epidemiology of TB among these populations and supports the previously documented need for more detailed analyses among members of different racial and ethnic groups.^8^ Detailed analyses such as this are particularly critical when exploring issues of health equity, as aggregating groups may hide key differences in a disease's incidence or experiences with risk factors.

These findings contribute to the body of evidence describing health disparities among NH/PI communities. To our knowledge, this is the first study to compare TB epidemiology between NH/PI persons born in the 50 states or Washington, DC and NH/PI persons born in the USAPI. This work highlights important differences in the epidemiology of TB by place of birth, demonstrating the need for TB interventions that appropriately address the needs of these different populations. Interventions that address social determinants of health, such as homelessness, may help reduce TB incidence among NH/PI populations. In addition, active TB screenings among persons born in the USAPI and efforts to increase TB treatment completion may help reduce TB transmission among NH/PI communities.

## Supplementary Material

Supplemental data

Supplemental data

Supplemental data

Supplemental data

Supplemental data

Supplemental data

## Abbreviations Used

CDC: Centers for Disease Control and Prevention
CI: confidence intervals
DC: District of Columbia
HHS: U.S. Department of Health and Human Services
IRR: incidence rate ratio
NH/PI: Native Hawaiian and Other Pacific Islander
NTSS: National Tuberculosis Surveillance System
PR: prevalence ratio
TB: tuberculosis
USAPI: U.S.-Affiliated Pacific Islands

## Notes

^†^
Afghanistan, Bangladesh, Bhutan, Burma, Cambodia, China, Hong Kong, India, Indonesia, Iran, Iraq, Israel, Japan, Jordan, Democratic People's Republic of Korea, Republic of Korea, Kazakhstan, Kyrgyzstan, Kuwait, Laos, Lebanon, Malaysia, Mongolia, Myanmar, Nepal, Pakistan, Philippines, Saudi Arabia, Singapore, Sri Lanka, Syria, Taiwan, Thailand, Turkey, United Arab Emirates, Uzbekistan, Vietnam, and Yemen.

^‡^
Includes patients who had immunosuppression due to either a medical condition or medication, or immunosuppressive therapy, excluding diabetes mellitus, end-stage renal disease, HIV/AIDS, and patients who had recently received, or were receiving, TNF-α antagonist therapy at the time of TB diagnosis.
