# Supplementary material for: Tuberculosis Among Native Hawaiian and Other Pacific Islander Persons: United States and U.S.-Affiliated Pacific Islands, 2010–2019
Source: Health Equity. 2022 Jun 27;6(1):476–84. doi: 10.1089/heq.2022.0065 (PMC9257550; doi:10.1089/heq.2022.0065)
Supplement: Supplemental data [file Supp_FileS1.docx]

Supplementary File 1

Supplementary Methods

*Clinical and Behavioral Characteristics*

Patient clinical and behavioral characteristics were defined based on definitions in the Report of a Verified Case of Tuberculosis, the form used to report a case of TB to CDC.^1^ Extrapulmonary disease was defined as evidence of disease in any of the following anatomical sites: pleural space, lymphatic system, larynx, bone, genital tract, meninges, or peritoneum. Individuals with the site of disease indicated as “other” were also considered to have extrapulmonary disease. Though there is no one definition for excess alcohol use, the determination can be made off various criteria such as: participation in an alcohol treatment program, medical documentation of excess alcohol use or hospitalization for an alcohol-related medical condition, or more than one arrest for intoxication or drunk and disorderly behavior.^1^ Drug use within the past year was defined as any self-reported injection or non-injection drug use within the past year.

Our primary analysis used Asian persons born in Asia as the reference group. We chose this group for two reasons. First, historically, NH/PI persons and Asian persons were grouped together as one racial group in NTSS, a determination based on the historical definition from the Office of Management and Budget (OMB).^2^ The groups were separated into two categories by OMB in 1997^2^ and formally changed in NTSS in 2003.^3^ Using Asian persons as a reference allowed us to examine differences between two groups historically categorized as the same racial/ethnic group. Second, non-U.S.–born Asian persons had the highest incidence of TB compared to other racial and ethnic groups in 2019^4^. Comparing to only Asian persons born outside the U.S. allowed us to explore potential differences between groups with the highest TB incidence.

Our secondary analysis used non-Hispanic White persons born in the 50 U.S. states or D.C. as the reference group. Past studies evaluating TB risk factors among indigenous persons have used U.S.-born White persons as the reference,^5, 6^ so including this secondary analysis allows us to compare results more easily across studies. Additionally, as U.S.-born White persons had the lowest incidence of TB in 2019^4^, using this group as the reference provides an important comparison to explore potential health disparities.

*Population estimates*

Population denominator estimates used to calculate crude annual TB incidence rates (per 100,000 persons) during 2010–2019 were generated using the following data sources and methods.

We obtained data from The U.S. Census Bureau’s American Community Survey (ACS).^7^ These data were the annual number of single race, non-Hispanic NH/PI persons who reported being born in any of the 50 states or D.C.; single race, non-Hispanic White persons born in any of the 50 states or D.C.; and single race, non-Hispanic Asian persons born in Asia. Countries included as part of Asia as defined by the ACS were Afghanistan, Bangladesh, Bhutan, Cambodia, China, Hong Kong, India, Indonesia, Iran, Iraq, Israel, Japan, Jordan, Korea (includes North and South Korea), Kazakhstan, Kyrgyzstan, Kuwait, Laos, Lebanon, Malaysia, Mongolia, Myanmar (including persons born in Burma), Nepal, Pakistan, Philippines, Saudi Arabia, Singapore, Sri Lanka, Syria, Taiwan, Thailand, Turkey, United Arab Emirates, Uzbekistan, Vietnam, and Yemen.^7^

The 2010 U.S. Census Bureau’s decennial census of Guam,^8^ American Samoa,^9^ and the Commonwealth of the Northern Mariana Islands^10^ provided estimates of the number of NH/PI persons who reported being born in each of those three areas. For each area, we used these values to estimate the proportion of the total population that was native-born. We then applied those proportions to the total population for each area from the U.S. Census Bureau’s International Database.^11^

Censuses conducted in the Federated States of Micronesia,^12^ the Republic of the Marshall Islands,^13^ and the Republic of Palau^14^ in 2010, 2011, and 2015, respectively, provided the estimated proportion of each area’s population that was native born; we assumed that persons who reported being born in these jurisdictions were NH/PI persons. We then applied these proportions to the total population for each area from the United Nations 2019 World Population Prospects.^15^

References

**1.** Centers for Disease Control and Prevention. Report of Verified Case of Tuberculosis (RVCT) Instruction Manual In: U.S. Department of Health and Human Services, ed; 2009.

**2.** Revisions to the Standards for the Classification of Federal Data on Race and Ethnicity In: Office of Management and Budget, ed*.* Vol 62; 1997.

**3.** Centers for Disease Control and Prevention. Reported Tuberculosis in the United States, 2020. 2021. https://www.cdc.gov/tb/statistics/reports/2020/default.htm. Accessed March 21, 2022.

**4.** Deutsch-Feldman M, Pratt RH, Price S, Tsang CA, Self JL. Tuberculosis -- United States, 2020. *Morbidity and Mortality Weekly Report.* 2021;70(12):409-414.

**5.** Springer Y, Kammerer JS, Silk BJ, Langer AJ. Tuberculosis in Indigenous Persons -- United States, 2009-2019. *Journal of Racial and Ethnic Health Disparities* 2021.

**6.** Manangan L, Elmore K, Lewis B, et al. Disparities in tuberculosis between Asian/Pacific Islanders and non-Hispanic Whites, United States, 1993-2006. *International Journal of Tuberculosis and Lung Disease* 2009;13(9):1077-1085.

**7.** United States Census Bureau. American Community Survey (ACS) 1-year estimates <https://data.census.gov/cedsci/>. Accessed November 20, 2021.

**8.** United States Census Bureau. Cross Tabulations, 2010 Island Areas - Guam Dataset. 2010. <https://www.census.gov/data/datasets/2010/dec/guam.html>. Accessed February 4, 2022.

**9.** United States Census Bureau. Cross Tabulations, 2010 Island Areas - American Samoa Dataset. 2010. <https://www.census.gov/data/datasets/2010/dec/american-samoa.html>. Accessed February 4, 2022.

**10.** United States Census Bureau. Cross Tabulations, 2010 Island Areas - Commonwealth of the Northern Mariana Islands. 2010. <https://www.census.gov/data/datasets/2010/dec/cnmi.html>. Accessed February 4, 2022.

**11.** The U.S. Census Bureau. International Database. <https://www.census.gov/data-tools/demo/idb/#/country?COUNTRY_YEAR=2022&COUNTRY_YR_ANIM=2022>. Accessed March 17, 2021.

**12.** FSM Statistics Office. FSM Statistics. 2020. <https://www.fsmstatistics.fm/social/population-statistics/>. Accessed January 24, 2022.

**13.** Economic Policy Planning and Statistics Office. The RMI 2011 Census of Population and Housing Detail Tables. file:///C:/Users/qdx3/Downloads/17.%20Detail%20Tables.pdf.

**14.** Republic Of Palau National Government. 2015 Census of Population Housing and Agriculture for the Republic of Palau. In: Office of Planning and Statistics, ed; 2015.

**15.** United Nations Department of Economic and Social Affairs Population Division. World Population Prospects 2019. 2019. https://population.un.org/wpp/DataQuery/. Accessed March 17, 2021.
