# Supplementary material for: Tuberculosis Among Native Hawaiian and Other Pacific Islander Persons: United States and U.S.-Affiliated Pacific Islands, 2010–2019
Source: Health Equity. 2022 Jun 27;6(1):476–84. doi: 10.1089/heq.2022.0065 (PMC9257550; doi:10.1089/heq.2022.0065)
Supplement: Supplemental data [file Supp_TableS1.docx]

**Supplementary Table 1: Reported country of birth among Native Hawaiian and Other Pacific Islander persons born outside of the 50 states or the U.S.-Affiliated Pacific Islands**

| **Country of birth** |  | **Number of cases** |
| --- | --- | --- |
| Total |  | 341 |
| Country |  |  |
|  | Papau New Guinea | 10 |
|  | Kirbati | 12 |
|  | Fiji | 21 |
|  | Philippines | 30 |
|  | Tonga | 35 |
|  | Western Samoa | 20 |
|  | Other countries (N < 10 cases per country)^a^ | 32 |
|  | Missing | 181 |

^a^Includes: Cambodia, Christmas Island, Eritrea, Guadeloupe, Guyana, India, Indonesia, Isle of Man, Korea, Laos, Miscellaneous, Mongolia, Nauru, Nepal, Pakistan, Saudi Arabia, Solomon Islands, Taiwan, Thailand, Tokelau, Trinidad and Tobago, Tuvalu, Vanuatu, Vietnam.
