# Supplementary material for: Tuberculosis Among Native Hawaiian and Other Pacific Islander Persons: United States and U.S.-Affiliated Pacific Islands, 2010–2019
Source: Health Equity. 2022 Jun 27;6(1):476–84. doi: 10.1089/heq.2022.0065 (PMC9257550; doi:10.1089/heq.2022.0065)
Supplement: Supplemental data [file Supp_TableS2.docx]

**Supplemental Table 2: Annual case counts and incidence of TB disease (cases per 100,000 persons) and incidence among non-Hispanic, single race NH/PI^a^, Asian and White persons by place of birth, 2010–2019.** Associated population estimates and incidence rate ratios (IRR), using the Asian and White analytic groups as the references, are also provided.

| Year | Count or rate-related statistic | NH/PI 50 States | NH/PI USAPI | Asian | White |
| --- | --- | --- | --- | --- | --- |
| 2010 |  |  |  |  |  |
|  | Total number of cases | 19 | 471 | 2,966 | 1,392 |
|  | Estimated population size^b^ | 286,225 | 267,751 | 9,643,092 | 187,978,680 |
|  | Crude case rate^c^ | 6.6 | 175.9 | 30.8 | 0.7 |
|  | IRR (95% CI)^d^ | 0.2 (0.1–0.3) | 5.7 (5.2–6.3) | Reference | --- |
|  | IRR (95% CI)^d^ | 9.0 (5.7–14.1) | 237.6 (214.0–263.7) | -- | Reference |
| 2011 |  |  |  |  |  |
|  | Total number of cases | 16 | 358 | 2,969 | 1,280 |
|  | Estimated population size^b^ | 284,651 | 268,152 | 9,911,331 | 188,000,565 |
|  | Crude case rate^c^ | 5.6 | 133.5 | 30.0 | 0.7 |
|  | IRR (95% CI)^d^ | 0.2 (0.1–0.3) | 4.5 (4.0–5.0) | Reference | -- |
|  | IRR (95% CI)^d^ | 8.3 (5.0–13.5) | 196.1 (174.4–220.5) | -- | Reference |
| 2012 |  |  |  |  |  |
|  | Total number of cases | 8 | 391 | 2,880 | 1,235 |
|  | Estimated population size^b^ | 300,671 | 269,986 | 10,216,763 | 188,161,770 |
|  | Crude case rate^c^ | 2.7 | 144.8 | 28.2 | 0.7 |
|  | IRR (95% CI)^d^ | 0.1 (0.0–0.2) | 5.1 (4.6–5.7) | Reference | -- |
|  | IRR (95% CI)^d^ | 4.1 (1.5–7.2) | 220.7 (196.9–247.2) | -- | Reference |
| 2013 |  |  |  |  |  |
|  | Total number of cases | 8 | 334 | 2,892 | 1,065 |
|  | Estimated population size^b^ | 310,611 | 270,790 | 10,473,469 | 188,152,481 |
|  | Crude case rate^c^ | 2.6 | 123.3 | 27.6 | 0.6 |
|  | IRR (95% CI)^d^ | 0.1 (0.0–0.2) | 4.5 (4.0–5.0) | Reference | -- |
|  | IRR (95% CI)^d^ | 4.6 (1.7–8.1) | 217.9 (192.7–246.4) | -- | Reference |
| 2014 |  |  |  |  |  |
|  | Total number of cases | 20 | 416 | 2,889 | 938 |
|  | Estimated population size^b^ | 318,852 | 271,503 | 10,955,174 | 188,198,384 |
|  | Crude case rate^c^ | 6.3 | 153.2 | 26.4 | 0.5 |
|  | IRR (95% CI)^d^ | 0.2 (0.2–0.4) | 5.8 (5.2–6.4) | Reference | -- |
|  | IRR (95% CI)^d^ | 12.6 (8.1–19.6) | 307.4 (273.9–345.0) | -- | Reference |
| 2015 |  |  |  |  |  |
|  | Total number of cases | 25 | 352 | 3,115 | 969 |
|  | Estimated population size^b^ | 313,775 | 273,131 | 11,428,525 | 188,138,399 |
|  | Crude case rate^c^ | 8.0 | 128.9 | 27.3 | 0.5 |
|  | IRR (95% CI) | 0.3 (0.2–0.4) | 4.7 (4.2–5.3) | Reference | -- |
|  | IRR (95% CI) | 15.5 (10.4–23.0) | 250.2 (221.5–282.7) | -- | Reference |
| 2016 |  |  |  |  |  |
|  | Total number of cases | 23 | 407 | 3,018 | 900 |
|  | Estimated population size^b^ | 332,161 | 274,615 | 11,512,744 | 187,980,642 |
|  | Crude case rate^c^ | 6.9 | 148.2 | 26.2 | 0.5 |
|  | IRR (95% CI)^d^ | 0.3 (0.2–0.4) | 5.7 (5.1–6.3) | Reference | -- |
|  | IRR (95% CI)^d^ | 14.5 (9.6–21.9) | 309.6 (275.4–348.0) | -- | Reference |
| 2017 |  |  |  |  |  |
|  | Total number of cases | 34 | 437 | 3,097 | 782 |
|  | Estimated population size^b^ | 336,056 | 275,180 | 12,002,617 | 187,734,968 |
|  | Crude case rate^c^ | 10.1 | 158.8 | 25.8 | 0.4 |
|  | IRR (95% CI)^d^ | 0.4 (0.3–0.6) | 6.2 (5.6–6.8) | Reference | -- |
|  | IRR (95% CI)^d^ | 24.3 (17.2–34.2) | 381.2 (339.2–428.6) | -- | Reference |
| 2018 |  |  |  |  |  |
|  | Total number of cases | 31 | 554 | 3,052 | 808 |
|  | Estimated population size^b^ | 334,289 | 276,668 | 12,020,706 | 187,492,959 |
|  | Crude case rate^c^ | 9.3 | 200.2 | 25.4 | 0.4 |
|  | IRR (95% CI)^d^ | 0.4 (0.3–0.5) | 7.9 (7.2–8.6) | Reference | -- |
|  | IRR (95% CI)^d^ | 21.5 (15.0–30.8) | 464.7 (417.1–517.7) | -- | Reference |
| 2019 |  |  |  |  |  |
|  | Total number of cases | 21 | 434 | 3,023 | 750 |
|  | Estimated population size^b^ | 343,692 | 278,024 | 12,123,548 | 187,364,728 |
|  | Crude case rate^c^ | 6.1 | 156.1 | 24.9 | 0.4 |
|  | IRR (95% CI)^d^ | 0.3 (0.2–0.4) | 6.3 (5.7–6.9) | Reference | -- |
|  | IRR (95% CI)^d^ | 15.3 (9.9–23.6) | 390.0 (346.5–438.9) | -- | Reference |

^a^NHPI = Native Hawaiian or Other Pacific Islander

^b^Population counts based on the United States Census Bureau American Community Survey (ACS) 5-year estimates (using the public use microdata sample (PUMS) data and person-weight weighting), U.S. Census Bureau’s International Database, and the United Nations World Population Prospects for the NH/PI 50 states, Asian, and White analytic groups, and the U.S. Census Bureau's decennial census and internally conducted censuses for the NH/PI USAPI group. See Supplemental Text for additional details regarding the population estimates.

^c^Calculated as cases per 100,000 persons.

^d^IRR = incidence rate ratio; CI = confidence interval, calculated using the normal approximation (Wald) method when the numerator for the non-reference group IRR was >10 and otherwise using a bootstrap method with 10,000 replicates. IRR and CI calculated using unrounded figures.
