# Supplementary material for: Tuberculosis Among Native Hawaiian and Other Pacific Islander Persons: United States and U.S.-Affiliated Pacific Islands, 2010–2019
Source: Health Equity. 2022 Jun 27;6(1):476–84. doi: 10.1089/heq.2022.0065 (PMC9257550; doi:10.1089/heq.2022.0065)
Supplement: Supplemental data [file Supp_TableS3.docx]

**Supplementary Table 3: Characteristics of NH/PI TB patients and Asian TB patients aged ≥ 15 years, stratified by place of birth, 2010–2019.** These data were used to generate Figure 4.

| **Characteristic** |  | **NH/PI 50 states^a^** | **NH/PI USAPI^a^** | **Asian^a^** |
| --- | --- | --- | --- | --- |
| Total number of cases in patients aged ≥15 years |  | 95 | 3,157 | 29,530 |
| Excess alcohol use within past year |  |  |  |  |
|  | Yes | 14 | 317 | 993 |
|  | No | 80 | 2,793 | 28,241 |
|  | Unknown/not reported | 1 | 47 | 296 |
|  | PR (95% CI)^b^ | 4.4 (2.7–7.1) | 3.0 (2.7–3.4) | Reference |
| Drug use within past year^c^ |  |  |  |  |
|  | Yes | 9 | 138 | 282 |
|  | No | 84 | 2,978 | 28,944 |
|  | Unknown/not reported | 2 | 41 | 304 |
|  | PR (95% CI)^b^ | 10.0 (4.3–17.0) | 4.6 (3.8–5.6) | Reference |
| Primarily unemployed within past year |  |  |  |  |
|  | Yes | 20 | 1,329 | 5,324 |
|  | Other^d^ | 69 | 1,768 | 23,407 |
|  | Unknown/not reported | 6 | 60 | 799 |
|  | PR (95% CI)^b^ | 1.2 (0.8–1.8) | 2.3 (2.2–2.4) | Reference |
| Experienced homelessness within past year |  |  |  |  |
|  | Yes | 9 | 58 | 309 |
|  | No | 86 | 3,087 | 29,107 |
|  | Unknown/not reported | 0 | 12 | 114 |
|  | PR (95% CI)^b^ | 9.0 (3.9–15.2) | 1.8 (1.3–2.3) | Reference |
| Diabetes mellitus at time of diagnosis |  |  |  |  |
|  | Yes | 19 | 829 | 6,505 |
|  | No | 76 | 2,328 | 23,025 |
|  | PR (95% CI)^b^ | 0.9 (0.6–1.4) | 1.2 (1.1–1.3) | Reference |
| End-stage renal disease at time of diagnosis |  |  |  |  |
|  | Yes | 5 | 44 | 904 |
|  | No | 90 | 3,113 | 28,626 |
|  | PR (95% CI)^b^ | 1.7 (0.3–3.5) | 0.5 (0.3–0.6) | Reference |
| Immunosuppressed at time of diagnosis^e^ |  |  |  |  |
|  | Yes | 5 | 32 | 1,792 |
|  | No | 90 | 3,125 | 27,738 |
|  | PR (95% CI)^b^ | 0.9 (0.2–1.7) | 0.2 (0.1–0.2) | Reference |
| Smear positive or cavitary disease^f^ |  |  |  |  |
|  | Yes | 42 | 1,430 | 12,383 |
|  | No | 24 | 1,088 | 9,359 |
|  | PR (95% CI)^b^ | 1.1 (0.9–1.3) | 1.0 (1.0–1.0) | Reference |

^a^NH/PI=Native Hawaiian and Other Pacific Islander; NH/PI 50 states includes all single-race NH/PI persons who reported being born in the 50 U.S. states or Washington D.C.; NH/PI USAPI includes all single-race NH/PI persons who reported being born in the USAPI; Asian includes all single-race Asian persons who reported being born in Asia.

^b^PR=Prevalence ratio, calculated as the quotient of the total number of “Yes” responses and the sum of the “Yes” and “No” responses (i.e., excluding the Unknown/missing responses; CI=Confidence Interval, calculated using the normal approximation (Wald) method when the numerator for any group PR was >10 and otherwise using a bootstrap method with 10,000 replicates.

^c^Includes injection and non-injection drug use

^d^Other includes "Correctional facility employee"; "Health care worker"; "Migratory agricultural worker"; "Not seeking employment"; "Other"; "Retired."

^e^Includes patients who had immunosuppression due to either a medical condition or medication, or immunosuppressive therapy, excluding diabetes mellitus, end-stage renal disease, HIV/AIDS, and patients who had recently received, or were receiving, TNF-α antagonist therapy at the time of TB diagnosis.

^f^Yes includes pulmonary patients with positive sputum smear or evidence of cavitation on chest x-ray or computerized tomography (CT) scan. No includes patients with negative sputum smear and no evidence of cavitation. Patients missing information regarding x-ray findings were included in the “No” category if there was no evidence of cavitation by CT and vice versa (and the patient’s sputum smear was negative).
