# Supplementary material for: Tuberculosis Among Native Hawaiian and Other Pacific Islander Persons: United States and U.S.-Affiliated Pacific Islands, 2010–2019
Source: Health Equity. 2022 Jun 27;6(1):476–84. doi: 10.1089/heq.2022.0065 (PMC9257550; doi:10.1089/heq.2022.0065)
Supplement: Supplemental data [file Supp_FigS1.docx]

**Supplementary Figure 1: Prevalence ratios comparing frequency of selected characteristics among NH/PI^a^ TB patients (50 states and USAPI^b^) with White patients**. Prevalence ratio (PR) calculated as the proportion of NH/PI TB patients reporting each characteristic, stratified by place of birth, divided by the proportion of U.S. states-born White TB patients reporting each characteristic. See Supplemental Table 2 for additional details. Persons with unknown or missing information for a characteristic were excluded from the PR calculation for that characteristic. The dashed line represents a null value of PR = 1.


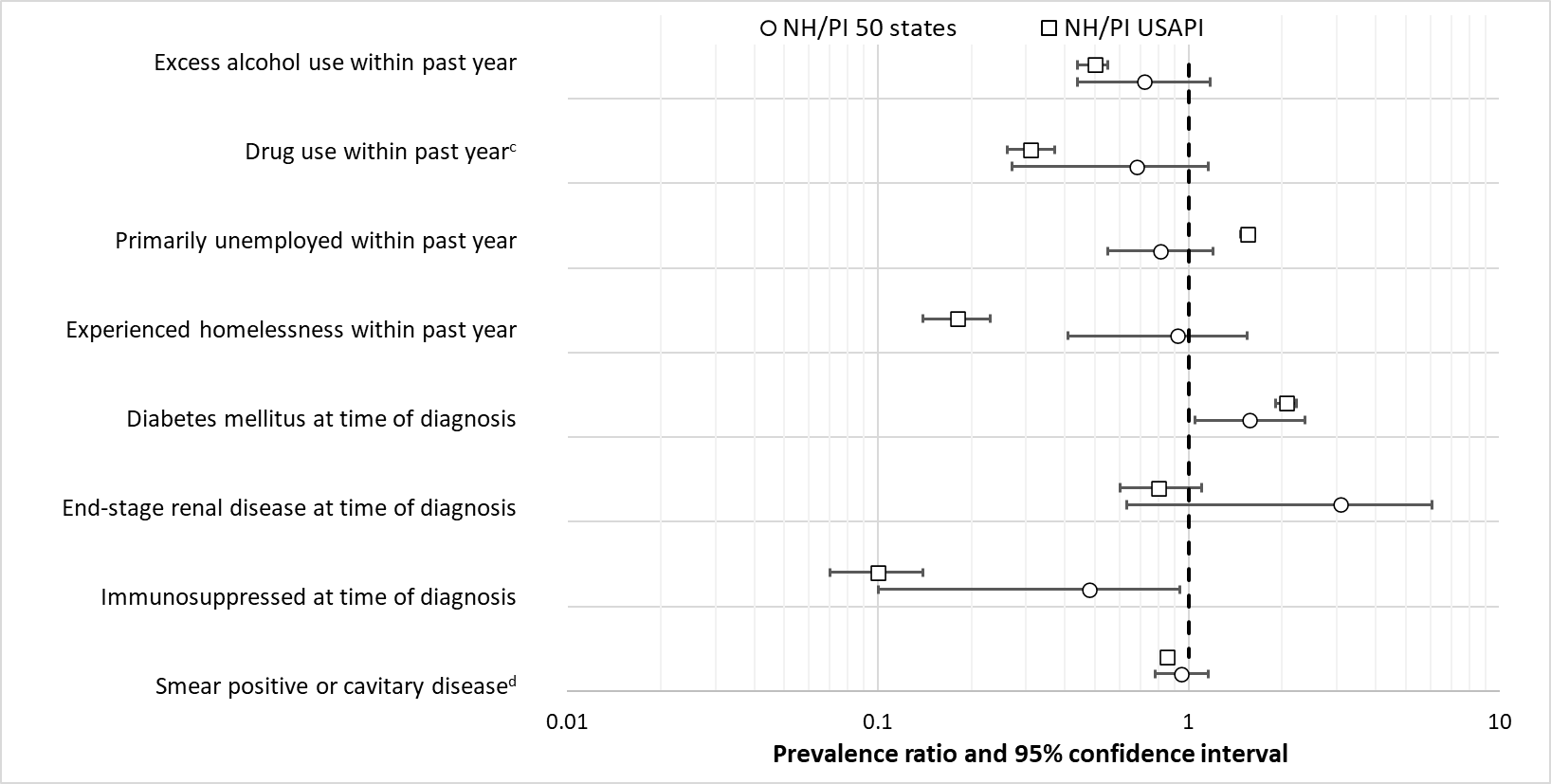


^a^NH/PI = Native Hawaiian and Other Pacific Islander

^b^USAPI = U.S.-Affiliated Pacific Islands

^c^includes both injection and non-injection drug use

^d^Smear = sputum smear; restricted patients with pulmonary disease
